# Intrinsic Polarization and Tunable Color of Electroluminescence from Organic Single Crystal-based Light-Emitting Devices

**DOI:** 10.1038/srep12445

**Published:** 2015-07-24

**Authors:** Ran Ding, Jing Feng, Wei Zhou, Xu-Lin Zhang, Hong-Hua Fang, Tong Yang, Hai-Yu Wang, Shu Hotta, Hong-Bo Sun

**Affiliations:** 1 State Key Laboratory on Integrated Optoelectronics, College of Electronic Science and Engineering, Jilin University, 2699 Qianjin Street, Changchun, 130012, People’s Republic of China; 2 College of Physics, Jilin University, 119 Jiefang Road, Changchun, 130023, People’s Republic of China; 3 Faculty of Engineering, Department of Electrical and Electronic Engineering, The University of Hong Kong, Pokfulam, Hong Kong; 4 Department of Macromolecular Science and Engineering, Graduate School of Science and Technology, Kyoto Institute of Technology, Matsugasaki, Sakyo-ku, Kyoto 606-8585, Japan

## Abstract

A single crystal-based organic light-emitting device (OLED) with intrinsically polarized and color-tunable electroluminescence (EL) has been demonstrated without any subsequent treatment. The polarization ratio of 5:1 for the transversal-electric (TE) and transversal-magnetic (TM) polarization at the emission peak of 575 nm, and 4.7:1 for the TM to TE polarization at the emission peak of 635 nm, respectively, have been obtained. The emitting color is tunable between yellow, yellow-green and orange by changing the polarization angle. The polarized EL and the polarization-induced color tunability can be attributed to the anisotropic microcavity formed by the BP3T crystal with uniaxial alignment of the molecules.

The research on organic light-emitting devices (OLEDs) has attracted much attention because of their potential applications in full-color flat panel displays and solid-state lighting^1^,^2^,^3^,^4^,^5^,^6^. Usually, electroluminescence (EL) from the OLEDs is unpolarized, which is beneficial to its general applications in lighting and displays. While highly polarized EL could also find their potential use in a wide range of applications, such as efficient backlight sources in liquid crystal displays, optical data storage, optical communication, and especially stereoscopic 3D display systems^7^,^8^,^9^. Polarized EL is usually realized by employing uniaxially oriented materials such as liquid crystalline polymers or oligomers incorporated in emissive layer^10^,^11^,^12^. Treatment methods are necessary to realize a uniaxially oriented film by using the uniaxially oriented materials, such as thermal annealing, vapor treatment, epitaxial growth or rubbed alignment layer^10^,^11^,^12^. Organic single crystals are ideal candidate for the polarized emission due to their intrinsic highly ordered structures with anisotropic properties, so that polarized emission is possible without any subsequent treatment. Highly polarized photoluminescence (PL) from the organic single crystal has been demonstrated^13^,^14^,^15^,^16^. Organic crystals have been widely used in optoelectronic devices such as optically pumped lasers, organic field-effect transistors (OFETs), light-emitting OFETs and photovoltaic cells, due to their features of highly ordered structure, high thermal stability, and high carrier mobility^17^,^18^,^19^,^20^,^21^,^22^,^23^,^24^. However, less attention has been paid to their use for the polarized EL, because of the poor performance of the organic single crystal-based OLEDs. In the former reports, the fragile crystal was usually transferred onto the pre-deposited metallic electrode and laminated by the weak van der Waals force, which resulted in a poor contact between the electrodes and crystal^16^,^25^. So there is a strong need of efficient and uniform carrier injection into the active layer of crystal for high performance and long-term stability of the crystal-based OLEDs.

Here we report on organic single-crystal-based OLEDs with intrinsic polarization and tunable color of EL. A single crystal of α,ω-Bis(biphenylyl)terthiophene (BP3T) is employed in the crystal-based OLEDs, which has been widely used and studied in the crystal-based OFETs^26^,^27^,^28^,^29^,^30^,^31^,^32^. An advanced technique of template stripping has been taken advantages in the fabrication of crystal-based OLEDs to solve the contact problem between the electrodes and the crystal^16^, which has been used to obtain ultrasmooth metallic films for plasmonics and metamaterials and realize flexible OLEDs and organic solar cells^33^,^34^,^35^,^36^. The bright and homogeneous surface EL emission can be realized due to the efficient and uniform carrier injection. Polarized EL can be easily obtained without any treatment. An anisotropic microcavity established by sandwiching the BP3T crystal between Ca/Ag and Au metallic electrodes is the main cause of polarized EL. The highly ordered molecular orientation induced birefringence with a large different refractive index between *a*- and *b*-axis of the BP3T crystal results in the anisotropic microcavity. There is a giant polarization splitting of EL from the BP3T-based OLEDs has been observed. The polarization ratio of 5:1 for the transversal-electric (TE) to transversal-magnetic (TM) polarization at the emission peak of 575 nm, and 4.7:1 for the TM to TE polarization at the emission peak of 635 nm, respectively, have been obtained. Furthermore the EL of the OLEDs exhibits a polarization-dependent emitting color. The emitting color of yellow with non-polarization, yellow-green with TE and orange with TM polarization can be achieved, and it is tunable by changing the polarization angle. The observation angle-resolved and polarization angle-resolved EL spectra are measured for the clarification of the mechanism of this polarized light emission. The polarized EL and the polarization-induced color tunability can be attributed to the anisotropic microcavity formed by the BP3T crystal with uniaxial alignment of the molecules.

## Results and Discussion

**Figure 1:**
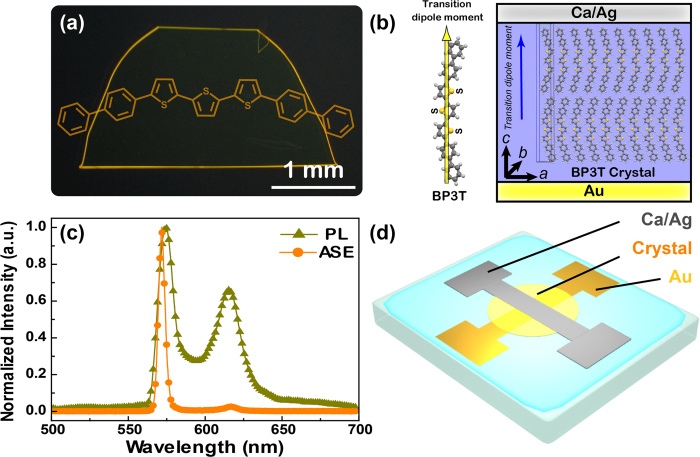
(**a**) Chemical structure of BP3T molecule and fluorescence photograph of BP3T crystal under the UV light irradiation. (**b**) The ordered BP3T molecular orientation in the OLEDs. (**c**) The PL and amplified spontaneous emission (ASE) spectra of the BP3T crystal. (**d**) Schematic representation of a BP3T single-crystal-based OLED.

**Figure 2:**
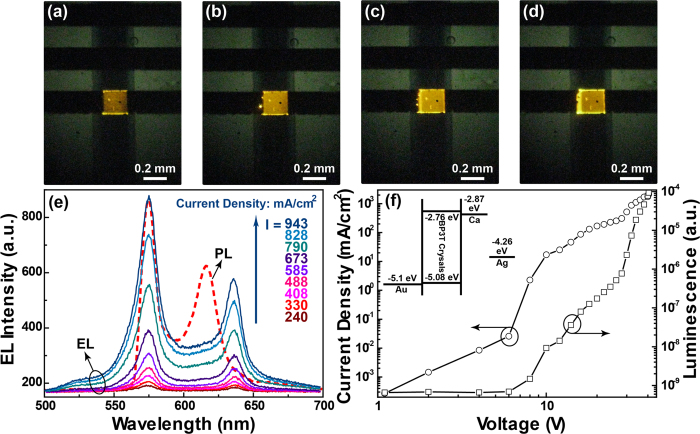
(**a**)–(**d**) Photographs of BP3T single-crystal-based OLEDs at different driving current of 165, 289, 477 and 643 mA/cm^2^ under operation. (**e**) The EL spectra detected at different driving current and the PL spectrum of BP3T single crystal as reference. (**f**) current density-voltage-luminance characteristics of the BP3T crystal-based OLEDs. Inset in f is the energy level diagram of the devices.

BP3T single crystal is chosen as the emitting material because of its high hole and electron mobilities of up to 1.64 and 0.17 cm^2^ V^−1^ s^−1^, and high luminescence efficiency of up to 80%^26^,^27^,^28^,^29^,^30^,^31^,^32^. Thin and flat BP3T single crystals can be grown by the improved physical vapor transport method^37^. BP3T crystals have been demonstrated the self-waveguided edge emission, so that light is confined within the crystal and the fringes are brighter than the edge (Fig. 1a). This well-defined PL can be ascribed to the transition dipole moments of organic single crystals in a nearly upright configuration (Fig. 1b)^38^,^39^. The PL spectrum is broad for low pump power whereas a strong narrow-band emission of amplified spontaneous emission (ASE) from BP3T single crystals will be observed at 575 nm by increasing the pump energy beyond a certain threshold (Fig. 1c)^26^,^27^,^28^,^29^,^30^,^31^,^32^. Here BP3T single crystal-based OLEDs are fabricated and characterized based on the technique of template stripping. The Au and Ca/Ag served as anode and cathode are both deposited onto the two sides of the organic single crystals by thermal evaporation, which ensures a compact contact between the electrodes and the crystal (Fig. 1d)^16^. As the grown condition is controlled precisely, the thickness of the BP3T is around 500 nm. The EL emission from the fringe of the OLEDs is brighter than that from the surface which is shown in Fig. 2a,b, which is coincident with that of the PL and can be attributed to the self-waveguide effect of the BP3T. Homogeneous surface emission can be observed at higher current density as can be seen from the bright picture in Supplementary Figure S1a, and the enlarge view of emission zone indicates a uniform carrier injection by the improved contact (Supplementary Figure S1b). The EL intensity from the device will follow with the increased current density as shown in Fig. 2e. The current density begins to increase at an onset voltage of around 1 V, and a rapid increase at voltage of 6 V indicating the current rectification behavior of the crystal-based OLED (Supplementary Figure S2a). Simultaneously, the EL intensity increases at the same voltage of 6 V observed from the current density-voltage-luminance characteristics (Fig. 2f). The low onset voltage can be ascribed to the improved contact between the electrodes and single crystals and the good energy level matching between the work function of metal electrodes deposited by thermal evaporation and the highest occupied molecular orbital and lowest unoccupied molecular orbital of BP3T single crystals (5.08 eV and 2.76 eV, respectively)^29^, which shows good performance of homogeneous luminance from the surface. And the energy level diagram of devices is shown in the inset of Fig. 2f. As the current density increases to be 778 mA/cm^2^, the luminance of the BP3T-based OLED can reach 5 cd/m^2^ (Supplementary Figure S2b). However, the external EL quantum efficiency (EQE) of the crystal-based OLEDs is still low, which may originate from a poor charge balance with different hole and electron mobilities of BP3T^16^,^25^,^29^. Herein, The reported hole mobility of p-type BP3T single crystals about 1.64 cm^2^ V^−1^ s^−1^ is much higher than the electron mobility of 0.17 cm^2^ V^−1^ s^−1^ 29. The luminance and EQE versus current density characteristics are shown in Supplementary Figure S2b. The EQE of crystal-based OLEDs is calculated by using the EL spectra and luminance according to the measured experiment data, as described in the Methods. Much effort is still needed to improve the EL efficiency of these crystal-based OLEDs in the future.

**Figure 3:**
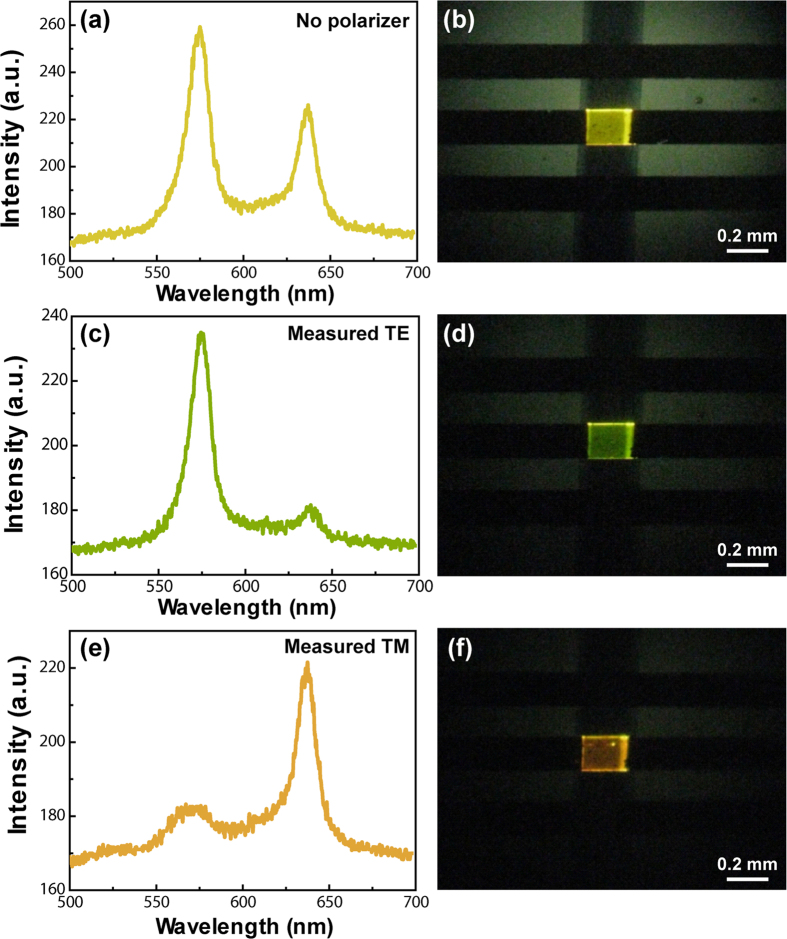
The optical polarization dependence of the EL spectra and the photographs of the BP3T crystal-based OLED with different polarization. Nonpolariztion (**a**), (**b**), TE (**c**), (**d**) and TM (**e**), (**f**) polarization, respectively.

A contrast polarizer is applied to analyze the polarization of the EL emission. The device emits yellow light without polarizer and the EL spectrum exhibits two major peaks at 575 and 635 nm, respectively (Fig. 3a,b). Then the two EL peaks can be separated by the polarizer and described as TE and TM polarization, respectively. The polarized EL spectra from the BP3T crystal-based OLEDs are measured at normal direction to the device surface and detected through a polarizer aligned parallel or perpendicular to the crystal plane, which are corresponding to TE and TM polarization, respectively. The TE and TM polarization with the electric and magnetic component are perpendicular with the crystal surface plane^40^,^41^. As the polarizer perpendicular to the crystal plane (TE polarization), the device shows yellow-green emission with the EL spectra dominated by the peak at 575 nm, while the peak of 635 nm is suppressed (Fig. 3c,d). Then the emission color can be changed to orange by tuning the polarizer parallel to the crystal plane (TM polarization), while the EL spectrum is dominated by the peak at 635 nm (Fig. 3e,f). The polarization ratio is estimated to be 5:1 for the TE to TM polarization at the spectrum peak of 575 nm, and 4.7:1 for the TM to TE polarization at the spectrum peak of 635 nm from emission, respectively.

**Figure 4:**
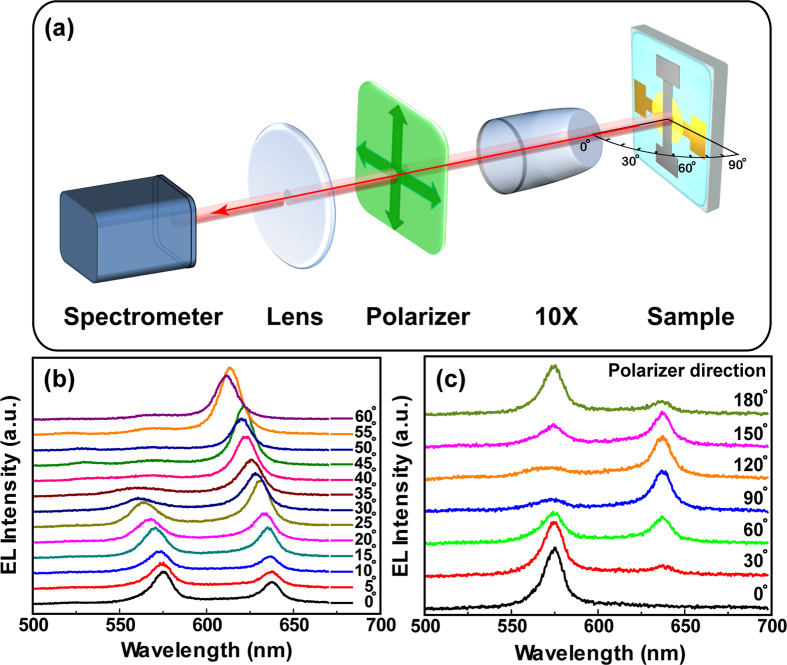
(**a**) A sketch of the experimental setup for the optical measurements, the observation angle variation is the angle to the normal of the sample (*c*-axis of the crystal) in the angle-resolved EL spectra measurement. (**b**) Angle-resolved EL spectra of BP3T single-crystal OLEDs by mounting the samples on a rotating holder. (**c**) Polarization angle-resolved EL spectra detected at normal incidence by varying the polarization direction.

In order to clarify the mechanism of this polarized light emission, the optical measurement system is set up for the observation angle-resolved and polarization angle-resolved EL spectra. The experiment setup is shown in Fig. 4a. The observation angle-resolved EL spectra are measured by rotating the observation angle to the *c*-axis of the crystal without polarizer. With the increasing of the observation angle from 0° to 60°, both emitting peaks centered at 575 nm and 635 nm shift towards short wavelength (Fig. 4b). The peak at the short wavelength moves out of the emission wavelength region of the crystal and disappears, when the observation angle is increased to 30°. On the basis of the template stripping technique, the anode and cathode of Au and Ca/Ag serving as reflecting mirrors are deposited on both sides of the organic single crystal. Then an anisotropic microcavity is therefore established by sandwiching the crystal between the metallic electrodes. Therefore, the blue shift of the emission peaks is caused by the microcavity effect. In the case of the polarization angle-resolved EL spectra (Fig. 4c) detected by varying the polarization direction of the polarizer, the polarization angle corresponds to the angle between the collection polarizer and the centre axis which is parallel to the OLEDs. By the rotation of the polarizer, the EL spectra can be changed from orange to yellow-green and yellow. The polarized EL and tunable emission color from the BP3T-based OLEDs can be attributed to the large birefringence difference between *a*-axis and *b*-axis of the crystal induced by the uniaxially alignment of the BP3T molecules^42^,^43^,^44^,^45^,^46^. The two EL peaks at 575 and 635 nm observed in the TE and TM polarized spectra correspond to the resonance of TE and TM modes in the anisotropic microcavity, respectively^47^,^48^,^49^. The TE and TM cavity modes exhibit a giant polarization splitting of around 60 nm which is much broader than the BP2T crystal-based OLEDs^16^. The polarization splitting is broad enough to tune the color emission from yellow-green to orange observed from the OLED due to a big difference of the refractive index between *a*-axis and *b*-axis of BP3T single crystals. There is also a giant polarization splitting observed from the polarized PL spectra measured with the crystal-based OLEDs (Supplementary Figure S3). Furthermore, the resonant wavelength in the microcavity will be determined by the total optical length depending on the thickness of crystals^16^. A comparison of different polarized EL spectra based on different crystal thickness is shown in the Supplementary Figure S4. The two cavity peaks will shift towards short wavelength when the thickness of crystals employed in the OLEDs decreases from 510 to 500 nm.

**Figure 5:**
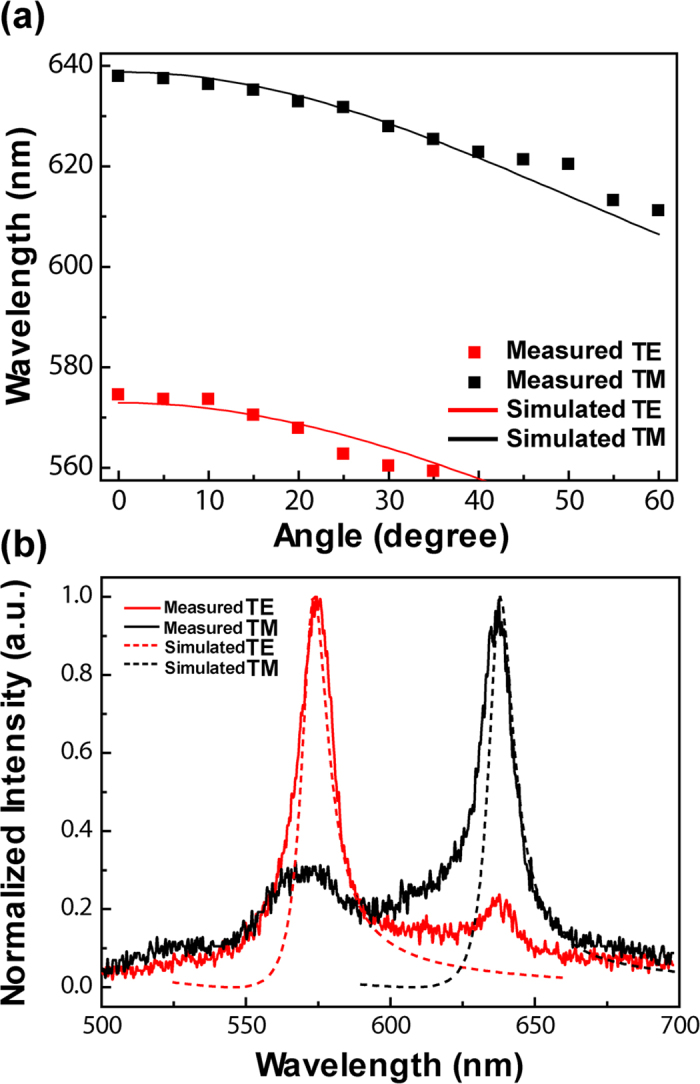
(**a**) TE and TM dispersion curves obtained from the angle-resolved EL measurements (squares) and the calculated results by the transfer matrix method (solid). (**b**) The comparison between the measured EL (solid) and calculated absorption (dash) spectra with TE and TM polarization.

The optical modes supported by the BP3T-based OLEDs can be established through the theoretical calculation on the absorption spectra by using the transfer matrix method^50^,^51^,^52^. The BP3T belongs to the thiophene-phenylene co-oligomers, which exhibits highly optical anisotropy due to the uniaxial molecule orientation. The anisotropic refractive indices for *a*-axis and *b*-axis of the BP3T single crystal are estimated to be 1.84 and 1.63, respectively, which are in good agreement with the calculation from the measured EL spectra. Then the different refractive indexes are employed in the transfer matrix method to simulate the optical modes. Figure 5a displays the dispersion relations for the TE and TM modes obtained from both the experimentally measured angle-resolved EL and the calculated absorption spectra by the transfer matrix method. With increased incident angle, the absorption peak shifts to blue in accordance with the dispersion relation of propagation constant *κ*_*a*_ and *κ*_*i*_, which coincides with that of the measured EL spectra. The experimental measured EL spectra and the calculated absorption spectra for the TE and TM modes at normal direction show consistent peak wavelength with each other as can be seen in Fig. 5b which confirms the origination of polarized EL spectrum peaks is from the microcavity modes. As a whole, due to this consistence, both the observed polarized EL and the giant polarization splitting can be attributed to the anisotropic microcavity from these crystal-based OLEDs.

## Conclusion

In summary, intrinsically polarized and color-tunable BP3T single-crystal-based OLEDs have been demonstrated in this paper. We observed a giant polarization splitting of the polarized EL spectra over 60 nm from BP3T-based OLEDs. The polarized EL exhibits a polarization ratio of 5:1 for the TE to TM polarization at the spectrum peak of 575 nm, and 4.7:1 for the TM to TE polarization at the spectrum peak of 635 nm from the emission, respectively. The emitting color is tunable between yellow, yellow-green and orange by tuning the polarization angle through a polarizer, ascribed to this giant polarization splitting. Based on the analysis of the observation angle-resolved and polarization angle-resolved EL spectra, the anisotropic microcavity caused by the birefringent BP3T crystals is confirmed as the main reason of the polarized EL and the polarization-induced color tunability. The exploration of the polarized and color-tunable EL from these crystal-based OLEDs is important for promoting their further applications.

## Methods

Growth of Organic Single Crystals: BP3T material was supplied by Kyoto Institute of Technology (KIT) with 99% pure. The material was not further purified before growth. Then the single crystals were grown by a method of horizontal physical vapor transport method with improved condition^37^. Neutral aluminum oxide was added into material to regulate the vapor saturation to an appropriate value. The crystals were found to be large, thin, and flat with growth times of 1–2 h. The sublimation temperature of 405 °C and growth temperature of 365 °C were controlled at the inner glass cylinders with a typical argon gas flow rate of 40 mL min^−1^. The thickness of the crystals can reach around 500 nm. And the size of crystals can be 3 mm × 3 mm which was large enough for the crystal-based OLED fabrication. The same size of BP2T single crystals have been employed in the device according to previous reports^16^. Although the specific parameters may be different from instrument to instrument, the principle of crystal growth remains the same.

Single Crystal-based OLEDs Fabrication and Evaluation: The Si substrates containing a silicon oxide dielectric with a thickness of 300 nm were in turn cleaned by acetone, ethanol and deionized water, at last dried with quick purged N_2_. Before any modification, the substrates were plasma cleaned for 5 min, octadecyltrichlorosilane (OTS) modification was carried out for about 4 h by vapor-deposition method. After the OTS-modification, the surface energy of Si/SiO_2_ substrate was 19.62 mJ/m^2^ ,which is much lower than that of the untreated Si/SiO_2_ substrate (47.73 mJ/m^2^). Then the substrate was equivalent to the hydrophobic surface which was benefitcial to stripping the single crystals off without any destruction. The surface energy was determined by the contact angle measurements according to the previous report^16^. Then the substrates were rinsed with chloroform, ethanol, deionized water by ultrasonic cleaning and dried with quick purged N_2_. At first, the BP3T crystals were laminated onto a pre-modified Si/SiO_2_ (300 nm) substrate. Then a thin metal template was used as the mask and a 100 nm thick Au anode was deposited by thermal evaporation. The third step was to produces a thin polymer solution layer on the silicon substrate with the pre-deposited crystal by compressing a microlitre polymer droplet (NOA63, Norland) with a smooth glass plate. The droplet was placed at around the centre of the substrate and then a 0.6 mm thick glass plate was adhered uniformly to the silicon substrate. The polymer layer spread across the entire glass-substrate interface. The sanwiched glass-polymer-silicon device was under the stress of the whole weight of the glass itself for 5–10 min at room temperature. And the polymer thickness ranged from 0.3 mm to 0.8 mm. Then the device was exposed to an ultraviolet light source at power of 125 W for 15 min. Finally, the glass was peeled off from the silicon substrate and a 10 nm Ca as a modified layer and a 25 nm Ag as cathode were evaporated onto the opposite surface of the BP3T crystal. The Ag metal layer was used to protect the Ca layer from being oxidized. Then the typical OLEDs based on single crystals were fabricated by above-mentioned processes under ambient conditions. All devices were fabricated with this sandwiched structure which the Au was used as anode and Ca/Ag as cathode. The thermal evaporation technique was used to grown the electrodes for both sides in a high vacuum condition of 5 × 10^−4^ Pa at 1 Å/s deposited rate. The active area of the device can be observed from the devices which was determined by the metal mask of 200 × 200 μm^2^. The detailed schematic of device fabrication is shown in the Supplementary Figure S5 16.

The current density-voltage-luminance characteristics of the devices were obtained at a voltage sweep measurement by a Photo Research PR-655 spectrophotometer with a Keithley 2400 programmable voltage-current source. The pictures of organic single crystals were taken by a Canon camera on BK-FL4 fluorescence microscope. The EL spectra were collected by a lens which focused the light on the end face of an optical fiber and analyzed by a mono-chromator coupled to a charge coupled device detector (CCD) and measured at steady state with constant current. The atomic force microscope (Digital Instruments Nanoscope IIIA) was used to determine the thicknesses and surface morphology for BP3T crystals which were measured in the tapping mode. The thickness of the BP3T crystal-based OLED employed for the measured polarized EL spectra and polarized EL photos of device in Fig. 3 was 510 nm (Supplementary Figure S6a). And the current density-voltage-luminance characteristics and emission photographs of device in Fig. 2a–d were based on a 500 nm thick OLED (Supplementary Figure S6b). From the Supplementary Figure S7a and b for the surface morphology of BP3T single crystal after stripping, there are no surface defects both at top and edge because of the non-destructive method of template stripping^16^.

Numerical Simulation for Optical Modes: Here the absorption spectra are calculated by the method of transfer matrix^50^,^51^,^52^. According to the transfer matrix method, the relative permittivity and permeability of the anisotropic active layer can be described as equation (1):
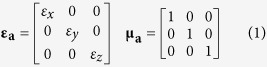
where z corresponds to the layer direction. And we assume an incidence coming from the active layer with a refractive index of *n*_*in*_ and an incident angle of *θ*. As the refractive indexes in crystals are anisotropic, the *n*_*in*_refers to the different indexes of *n*_*a*_ and *n*_*b*_along the *a-*axis and *b-*axis in the crystal plane, respectively. Then we simply take the normal TM-polarized case with electromagnetic components of *E*_*x*_, *E*_*z*_, and *H*_*y*_ for instance. The magnetic field can be described as 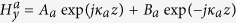 in the anisotropic active layer, where *A*_*a*_ and *B*_*a*_ are undetermined amplitude coefficients, and the *κ*_*a*_ is denoted 
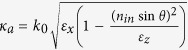
. And the magnetic field will be 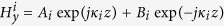 when the electromagnetic waves in the isotropic layer with a relative permittivity of *ε*_*i*_, and 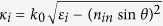. Then we can get the x-direction electric field by applying the relationship of 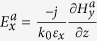 for waves in the anisotropic active layer and 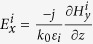 for waves in the isotropic layer, respectively. We apply the continuity of the tangential electromagnetic components (*E*_*x*_ and *H*_*y*_) in the layer boundary to obtain the transfer matrix between each adjacent layer. Then the undetermined amplitude coefficients *A*_*a*_, *B*_*a*_, *A*_*i*_, and *B*_*i*_ can be solved and the absorption spectra can be calculated by some further operations of the Poynting vectors.

External Quantum Efficiency Calculation: The EQE of crystal-based OLEDs (*η*_*ext*_) is calculated by using equation (2) as follow^53^:
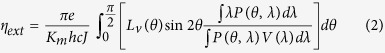
where *e* corresponds to the quantity of the electron charge, *K*_*m*_ is a conversion constant based on the maximum sensitivity of the eye (683 lm W^−1^), *h* is the Planck constant, *c* is the velocity of the light, *J* is the measured current density, *P(θ, λ)* is the relative spectral power distribution of the device at viewing angle *θ* corresponding to the EL spectral intensity distribution measured from EL spectra, the EL spectra at current of 778 mA/cm^2^ is shown in the inset of Supplementary Figure S2b, *V(λ)* is the normalized photopic spectral response function, and *L*_*v*_*(θ)* is the spectral luminance at *θ.*

## Additional Information

**How to cite this article**: Ding, R. *et al.* Intrinsic Polarization and Tunable Color of Electroluminescence from Organic Single Crystal-based Light-Emitting Devices. *Sci. Rep.* **5**, 12445; doi: 10.1038/srep12445 (2015).

## Supplementary Material

Supplementary Information
