# Supplementary material for: Intrinsic Polarization and Tunable Color of Electroluminescence from Organic Single Crystal-based Light-Emitting Devices
Source: Sci Rep. 2015 Jul 24;5:12445. doi: 10.1038/srep12445 (PMC4513548; doi:10.1038/srep12445)
Supplement: Supplementary Information [file srep12445-s1.doc]

Intrinsic Polarization and Tunable Color of Electroluminescence from Organic Single Crystal-based Light-Emitting Devices

Ran Ding^1^, Jing Feng^1^, Wei Zhou^1^, Xu-Lin Zhang^1^, Hong-Hua Fang^1^, Tong Yang^3^, Hai-Yu Wang^1^, Shu Hotta^4^ & Hong-Bo Sun^1,2^

^1^State Key Laboratory on Integrated Optoelectronics, College of Electronic Science and Engineering, Jilin University, 2699 Qianjin Street, Changchun, 130012, People’s Republic of China.

^2^College of Physics, Jilin University, 119 Jiefang Road, Changchun, 130023, People’s Republic of China.

^3^Faculty of Engineering, Department of Electrical and Electronic Engineering, The University of Hong Kong, Pokfulam, Hong Kong.

^4^Department of Macromolecular Science and Engineering, Graduate School of Science and Technology, Kyoto Institute of Technology, Matsugasaki, Sakyo-ku, Kyoto 606-8585, Japan.

Correspondence and requests should be addressed to Prof. Jing Feng ([jingfeng@jlu.edu.cn](mailto:jingfeng@jlu.edu.cn)) and Prof. Hong-Bo Sun ([hbsun@jlu.edu.cn](mailto:hbsun@jlu.edu.cn))

**Supporting Information**

**1. Bright picture and enlarge view of emission zone taken by the BP3T single-crystal-based OLEDs.**


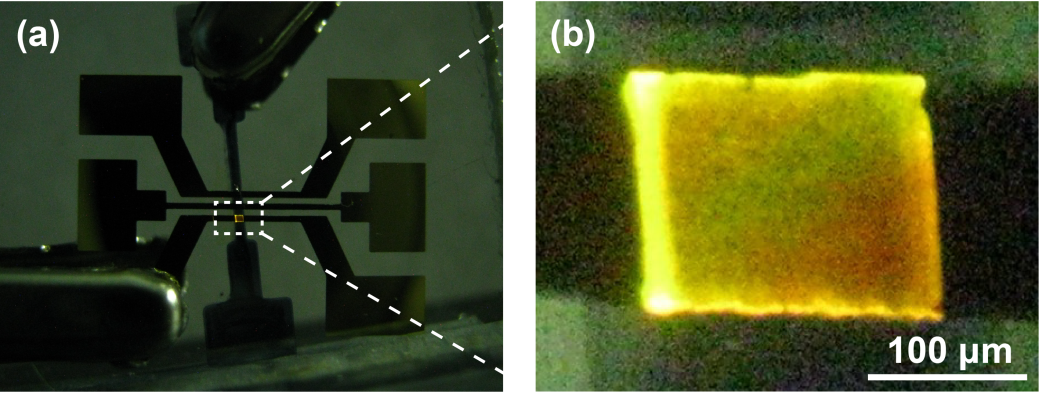


**Figure S1.** (a) Photograph of the operating BP3T-OLEDs under day light (b) Enlarge Photograph of emission zone.

**2. The I-V curve indicating the current rectification behavior of the BP3T crystal-based OLED and Current-density dependence on luminance and EQE.**


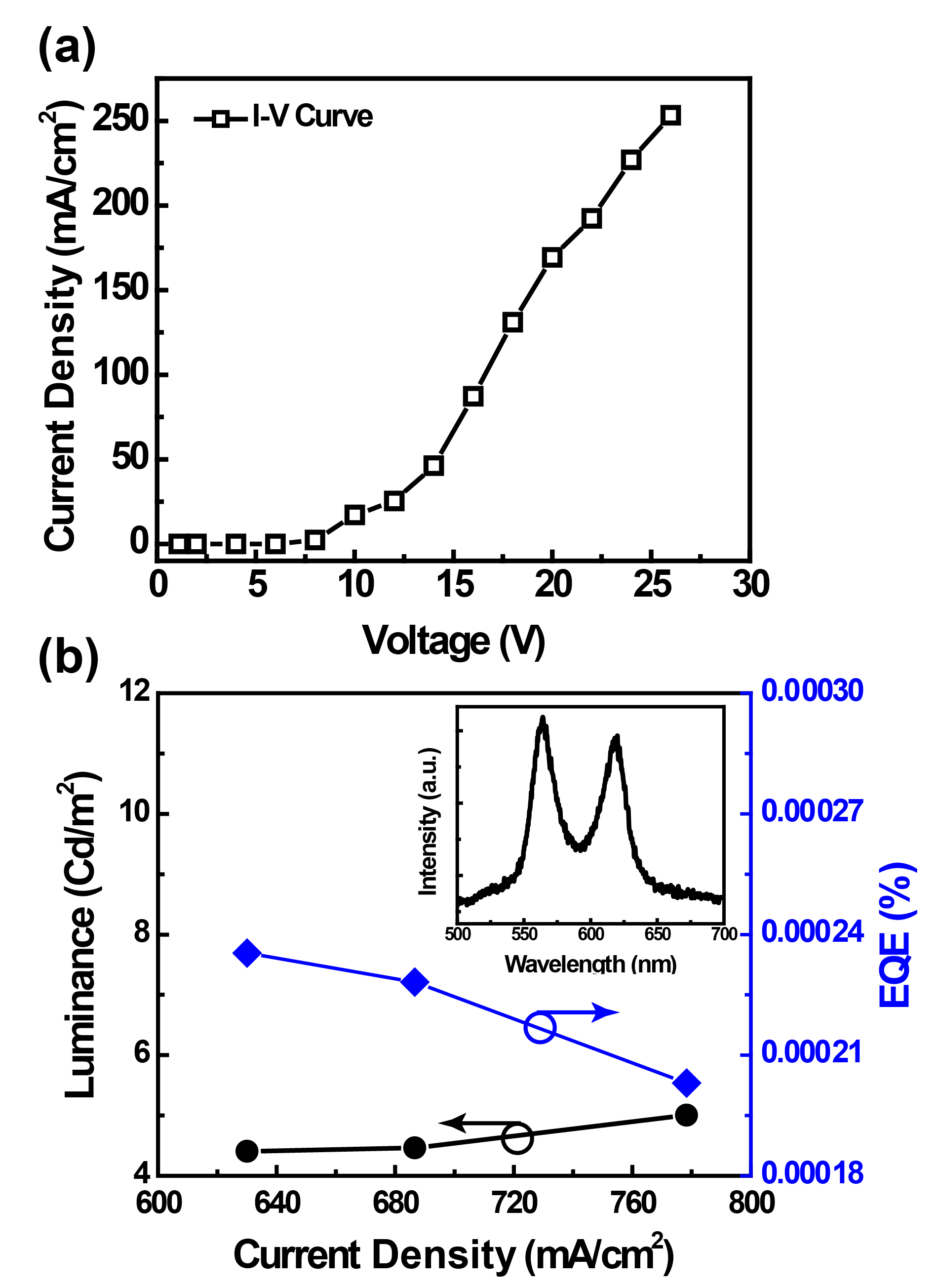


**Figure S2.** (a) I-V curve of a BP3T crystal-based OLED. (b) Current-density dependence on luminance and EQE. The inset shows a EL spectrum of the BP3T crystal-based OLED at current of 778 mA/cm^2^.

**3. The Non-polarized, TE polarized and TM polarized polarized PL spectra of BP3T crystal-based OLEDs.**


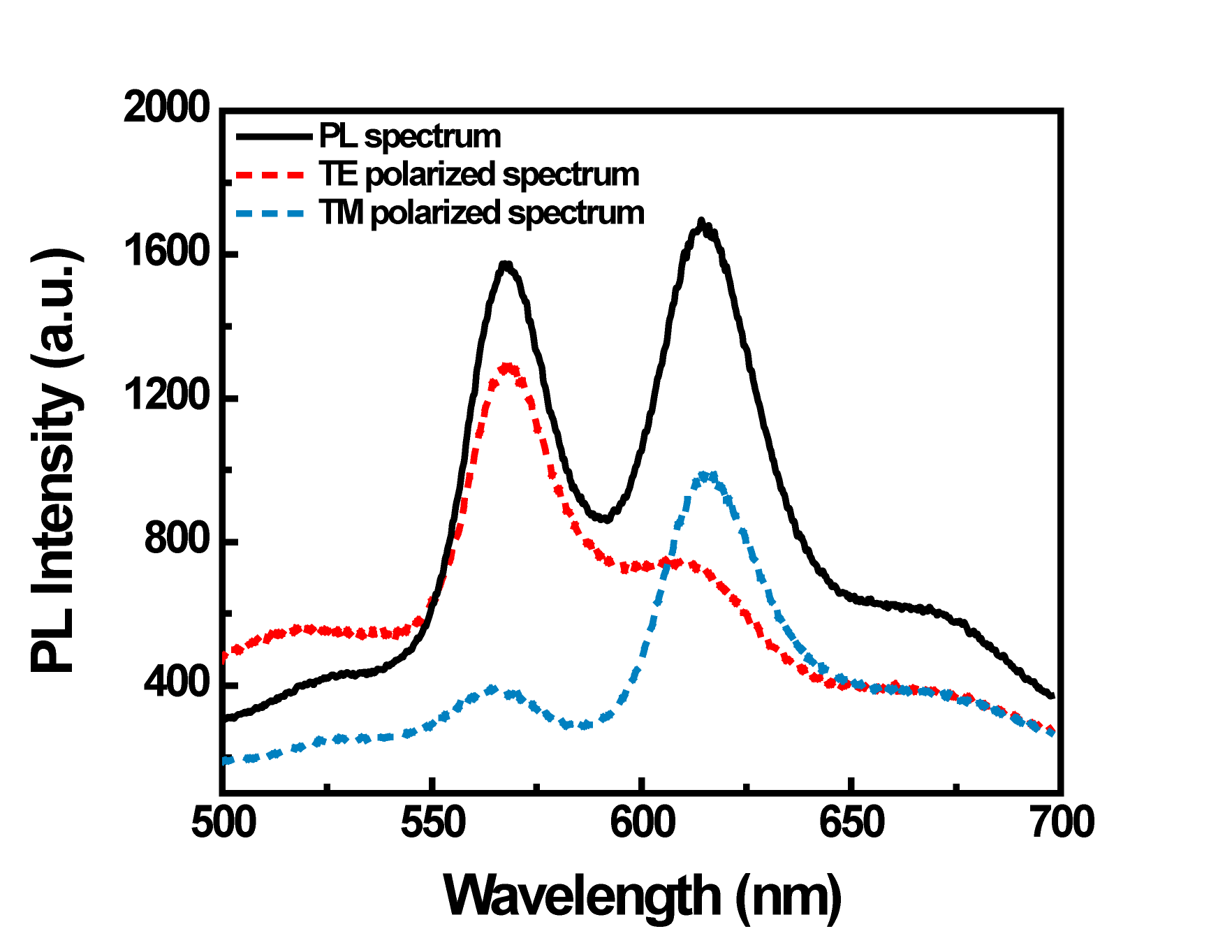


**Figure S3.** Non-polarized, TE polarized and TM polarized PL spectra of BP3T crystal-based OLEDs.

**4. Polarized EL spectra and polarization angle-resolved EL spectra from OLEDs with different thickness of BP3T single crystals**


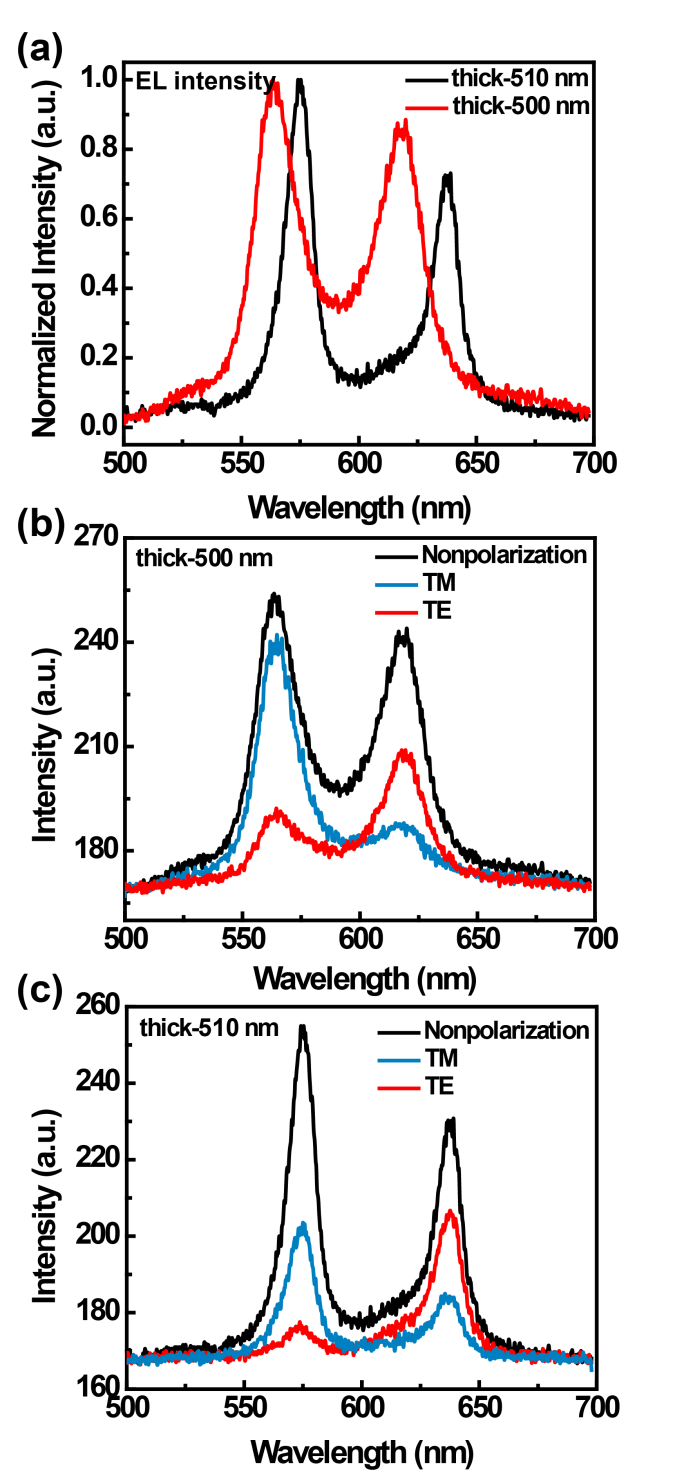


**Figure S4.** (a) Nonpolarized EL spectra from OLEDs with 500 and 510 nm thick BP3T single crystals, respectively. (b)-(c) TM and TE polarized EL spectra from OLEDs with 500 and 510 nm thick BP3T single crystals, respectively.

**5. The detailed schematic of device fabrication**


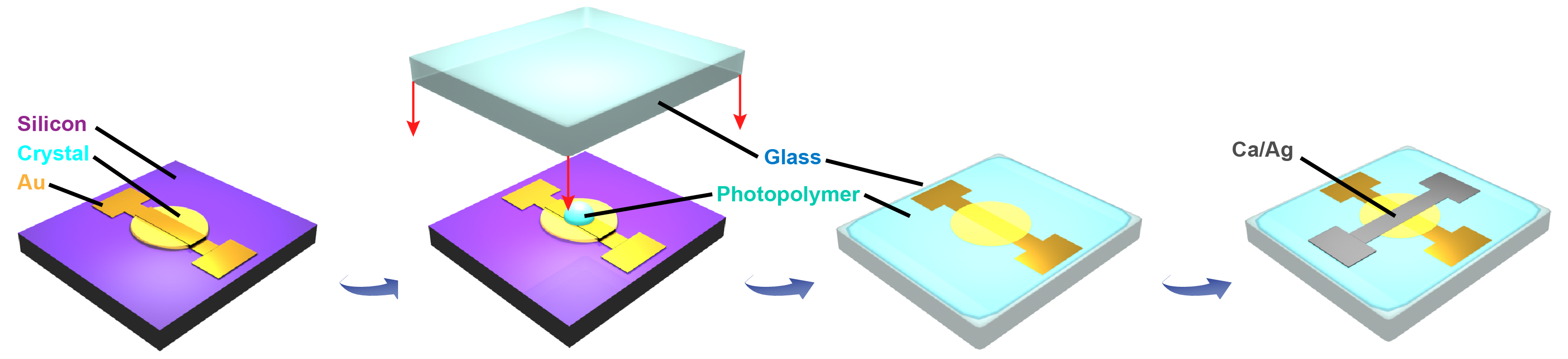


**Figure S5.** Schematic of fabrication process of template stripping technique for the crystal-based OLEDs. After the single crystal transferred onto the OTS-treated Si/SiO_2_ substrate, a 100 nm thick Au anode thermal evaporated onto the crystal surface. Then a droplet of photopolymer is placed at the center of the device and compressed by a flat glass to form a photopolymer layer at the interface. Thirdly, the photopolymer exposed to a UV light and peeled off from the OTS-treated Si/SiO_2_ substrate with the Au-coated crystal. Finally, a cathode with 10 nm Ca and 25 nm Ag is deposited onto the opposite side of the crystal.

**6. Atomic force microscopy (AFM) images of the crystal thickness**


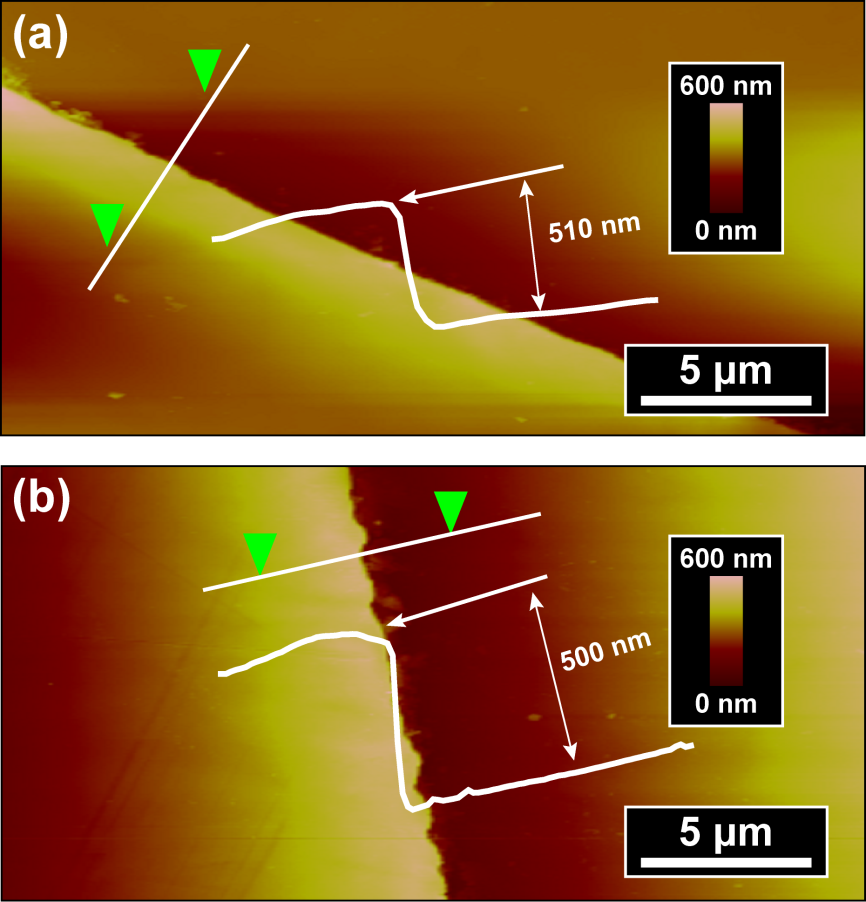


**Figure S6.** Atomic force microscopy (AFM) images of the crystal thickness employed in the fabrication of BP3T crystal based OLEDs.

**7. AFM images of the crystal surface after stripping.**


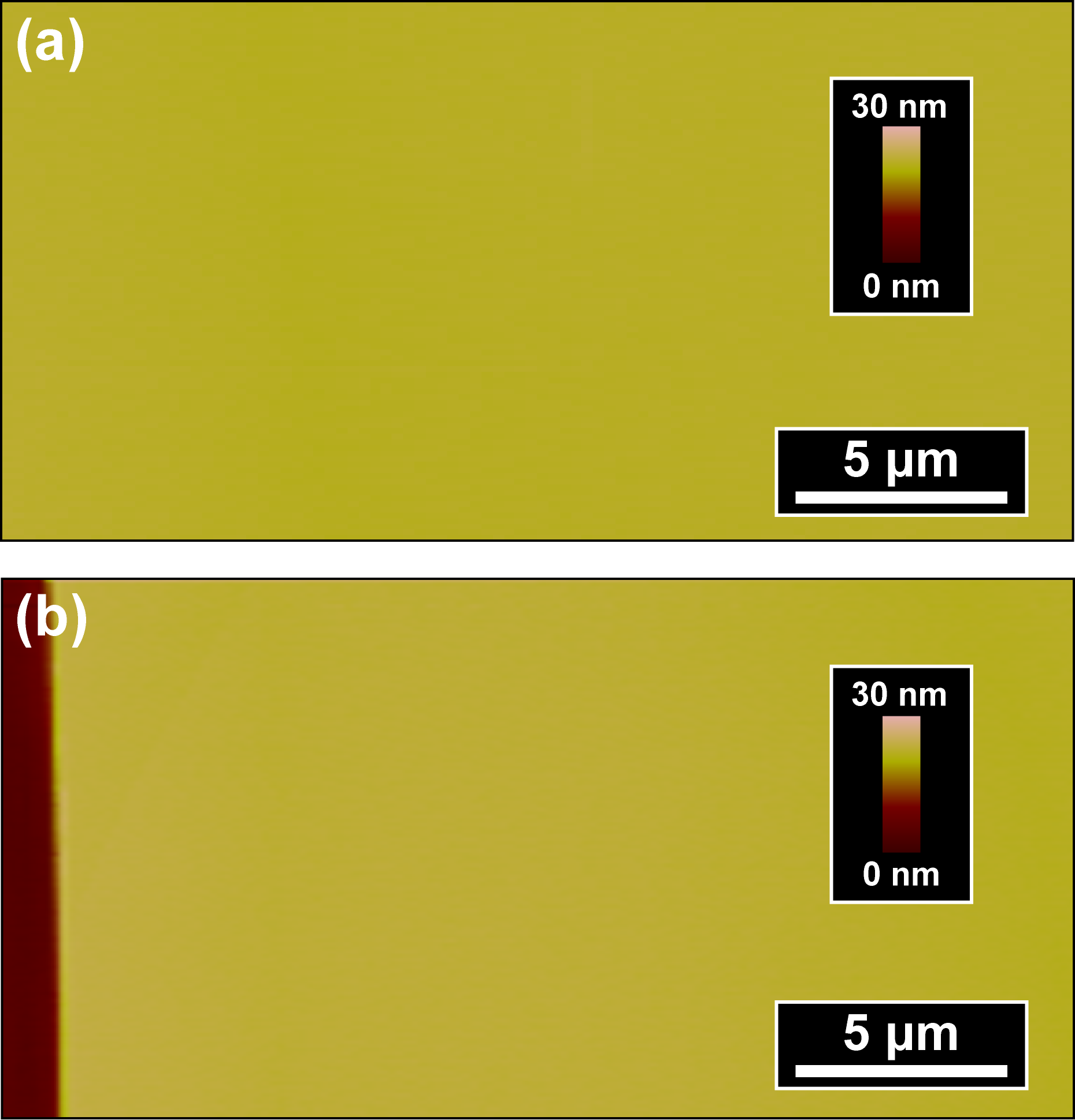


**Figure S7.** AFM images at top (a) and edge (b) of the crystal surface after stripping employed in the fabrication of BP3T crystal based OLEDs.
